# Morpho-agronomic evaluation of native maize races associated with Mexican tropical climate agroforestry systems

**DOI:** 10.1371/journal.pone.0269896

**Published:** 2022-06-14

**Authors:** Gregorio Hernández-Salinas, Filiberto Toledano-Toledano, Maximino Pérez-García, Oscar Valeriano Sánchez-Valera, Emmanuel de Jesús Ramírez-Rivera, Ricardo Serna-Lagunes, Mario Rocandio-Rodríguez, Rubén Purroy-Vásquez, Claudia Lorena Fernández-López, Fernando López-Morales, Juan Garduño-Espinosa

**Affiliations:** 1 Tecnológico Nacional de México, Instituto Tecnológico Superior de Zongolica, Zongolica, Veracruz, México; 2 Unidad de Investigación en Medicina Basada en Evidencias, Hospital Infantil de México Federico Gómez, México City, México; 3 Unidad de Investigación Sociomédica, Instituto Nacional de Rehabilitación Luis Guillermo Ibarra Ibarra, Tlalpan, México City, Mexico; 4 Biological Science and Agronomy Faculty, Universidad Veracruzana, Amatlán de los Reyes, Veracruz, México; 5 Instituto de Ecología Aplicada, Universidad Autónoma de Tamaulipas, Ciudad Victoria, Tamaulipas, México; 6 Universidad Politécnica de Huatusco, Huatusco, Veracruz, México; 7 Manejo Sostenible de Agroecosistemas, Centro de Agroecología, Instituto de Ciencias- Benemérita Universidad Autónoma de Puebla, San Pedro Zacachimalpa, Puebla, México; Central Research Institute for Dryland Agriculture, INDIA

## Abstract

Maize (*Zea mays* L.) is native to Mexico, in which wide genetic diversity can be found; however, maize is at risk of genetic erosion, and agroforestry systems (ASs) can be a strategy for conservation and sustainable use of this crop. The objective of this study was to evaluate the variation in the morpho-agronomic characteristics of three native maize races, Tuxpeño, Olotillo × Tuxpeño and Ratón × Tepecintle, cultivated in different AS in a tropical climate of Veracruz, Mexico, as well as its association with microclimatic conditions. In 2019, experiments were established in the localities La Gloria and La Luisa, Veracruz, where the three maize races are cultivated, in a randomized complete block design with three replications in a 3 × 4 factorial scheme (three native maize races and three AS arrrays, plus monoculture). Ten morpho-agronomic variables were recorded in each experiment and were analyzed by analysis of variance (ANOVA; Tukey’s post-hoc test, all *p* ≤ 0.05) and principal component analysis (PCA). Six morpho-agronomic characteristics showed significant differences for the race × system interaction. Consistently standing out both in the *Myroxylon* with 2.8 m × 2.0 arrays and in the monoculture was the Olotillo × Tuxpeño race, as there were no variations (*p* ≥ 0.05) in 50% of its morpho-agronomic characteristics. The first three PCs explained 87.7% of the cumulative variance, determined by five variables of the ears, three of the grain and plant height, which were associated with temperature; therefore, the microclimatic conditions of the studied ASs are associated with the morpho-agronomic characteristics of the native maize races. The results show that ASs could be a strategy for the conservation and use of native corn germplasm and could allow the diversification of sustainable production for rural farmers.

## Introduction

Approximately 7000 species of cultivated plants have been domesticated in Mesoamerica [1], but only 30 contribute approximately 90% of the global food security of the population [2]. Among them are wheat (*Triticum aestivum*), rice (*Oriza sativa*) and maize (*Zea mays*) [3]. In 2050, the world population is expected to reach 9 700 million, so a major goal is to ensure food security [3]. In addition, it is mandatory to preserve plant genetic resources under a sustainable agricultural system without compromising natural resources and environmental balance [4].

In Mexico, *Z*. *mays* is a native species that was domesticated and diversified by prehispanic cultures. It has high sociocultural and economic importance. Terán and Rasmussen [5] reported that maize had been cultivated by Mayan culture in milpa systems. Evidence of teosintle derivation has been found [6]. Additionally, 64 native maize races have been identified (17% of the total found on the North American continent). These races are distributed between a wide variety of climate and altitude conditions, from sea level to nearly 3 400 m [7].

The wide morpho-agronomic and genetic variation registered in native maize races from Mexico [8, 9] is being lost at rates of approximately 1.22 to 1.43 of native races per producer in 5-year intervals [10]. This implies genetic erosion of germplasm [11]. This can be due to several factors, such as no interest in culture [12], introduction of monocultures with improved varieties, low productivity and culture reconversion of sugar cane *(Saccharum* spp.) and people migration [11, 13, 14].

Faced with this loss of diversity of native maize races, ASs are part of a strategy for the conservation and sustainable use of plant genetic resources [15, 16]; furthermore, the combination of trees, bushes, crops, and animals integrates an economical and sustainable environmental option. This might benefit small producers in tropical regions [17]. However, due to the unknown morpho-agronomic variation of maize races under different AS conditions, the adoption level of this technology by farmers or other actors involved in this field of knowledge is low [16, 18].

Previous empirical evidence [19–24] has documented the viability of intercropping ASs with annual crops such as gramineous sugar cane (*Saccharum* spp.), wheat (*Triticum aestivum* Linn) and maize (*Z*. *mays*), which have C4 metabolism, and the same researchers have found differences in the morphological, agronomic and physiological characteristics.

Schwerz et al. [25] evaluated the leaf area index, dry matter, yield, juice volume, solar radiation interception, and efficient use of solar radiation in sugar cane (*Saccharum officinarum* L.) in AS arrays and found important differences in productive and physiological variables between the AS system and monoculture. They attributed this behavior to the tree canopy, interactions between plants, plantation spatial arrangement, microclimatic conditions, and competition for resources such as radiation. Caron et al. [22] studied morpho-productive and physiological aspects in *T*. *aestivum* cv. BRS Tarumã in AS arrays, in addition to incorporating monocultures, and found that a decrease in solar radiation affected the dried matter, kernel yield, leaf area index, phytochrome content, and photosynthetic rate of the cultivar itself. These researchers are in the process of evaluating other variables such as microclimatic conditions, plantation space arrangement, and the morphophysiological plasticity of genotypes.

Artru et al. [24] analyzed the artificial shading effect in wheat plants (*T*. *aestivum* cv. Edgard) where differences were found in dry matter, yield and its components, and the kernel protein concentration between treatments with and without artificial shading during plant phenological processes.

However, there is limited research on morpho-agronomic variation in *Z*. *mays* intercropped with ASs. Bertomeu [20] found that in maize hybrids, grain yield was less related to monoculture-grown trees because shading affects this variable. Nardini et al. [23] reported that the growth of hybrid maize plants (e.g., the leaf index area and net growth rate) was affected by tree shading, but when plants were cultivated under AS conditions, they showed a higher efficiency with respect to solar radiation than when they were grown in monocultures.

In Mexico, ASs have been studied from a conservation approach, particularly native biodiversity, and their relationship with the traditional knowledge of farmers [26, 27]. These researchers have emphasized that these farmers are immersed in a global economic market where conventional agriculture is promoted, as it is essential to incorporate traditional knowledge into public policies on the conservation of plant genetic resources. All of the above-described findings are evidence of the lack of knowledge about the morpho-agronomic variation of the maize races Tuxpeño, Olotillo × Tuxpeño, and Ratón × Tepecintle native to Mexico associated with AS schemes; therefore, generating this knowledge is critical to encourage *in situ* conservation strategies and the sustainable use of these races through the implementation of appropriate SA arrangements for each of them. In addition, with this information, planned decisions could be made in the transfer of this technology to small producers in rural areas of tropical areas to contribute to the diversification of their products, such as wood, grains and ecosystem services [16–18]. In this sense, the objectives of the present work were 1) to evaluate the variation in the morpho-agronomic characteristics of three races of maize native to Mexico, Tuxpeño, Olotillo × Tuxpeño and Ratón × Tepecintle, cultivated under different AS arrangements in a climate tropical and 2) to relate the morpho-agronomic variables of the three native maize races to the microclimatic conditions recorded in the AS arrangements.

## Materials and methods

### Collection and classification of genetic material

In April and May 2019, the first botanical exploration was carried out in three locations in Veracruz, Mexico (Table 1), to collect populations of native maize cultivated in the State of Veracruz [28]. Collection was made following Ortega-Paczka [29]. These collections were deposited in the germplasm bank of the Instituto Tecnológico Superior de Zongolica with corresponding passport data, and they were classified racially by the Mexican expert Rafael Ortega Paczka.

**Table 1:** Passport data of native maize races examined in this study.

Collection location	Race	Kernel color	North latitude	West longitude	Altitude (m)
La Gloria	Tuxpeño	White	18.2425	-96.4136	94
El Mirador	Olotillo × Tuxpeño	Purple	18.2309	-96.4357	101
Raya Caracol	Ratón × Tepecintle	Yellow	18.2301	-96.4147	95

### Study description

The study was performed in the following locations: La Gloria and la Luisa, municipality of Tezonapa, Veracruz (18.2425 LN, -96.4136 LW, at 94 m.a.s.l; 18.3420 LN, -96.4310 LW, at 180 m.a.s.l, respectively). Both locations have warm-wet climates (Am), rainy summers and an average annual temperature of 18°C and mean annual precipitation of 1259.3 mm, with Acrisol and Lixisol soils, respectively [30, 31].

### Establishment and management of experimental plots

**Fig 1:**
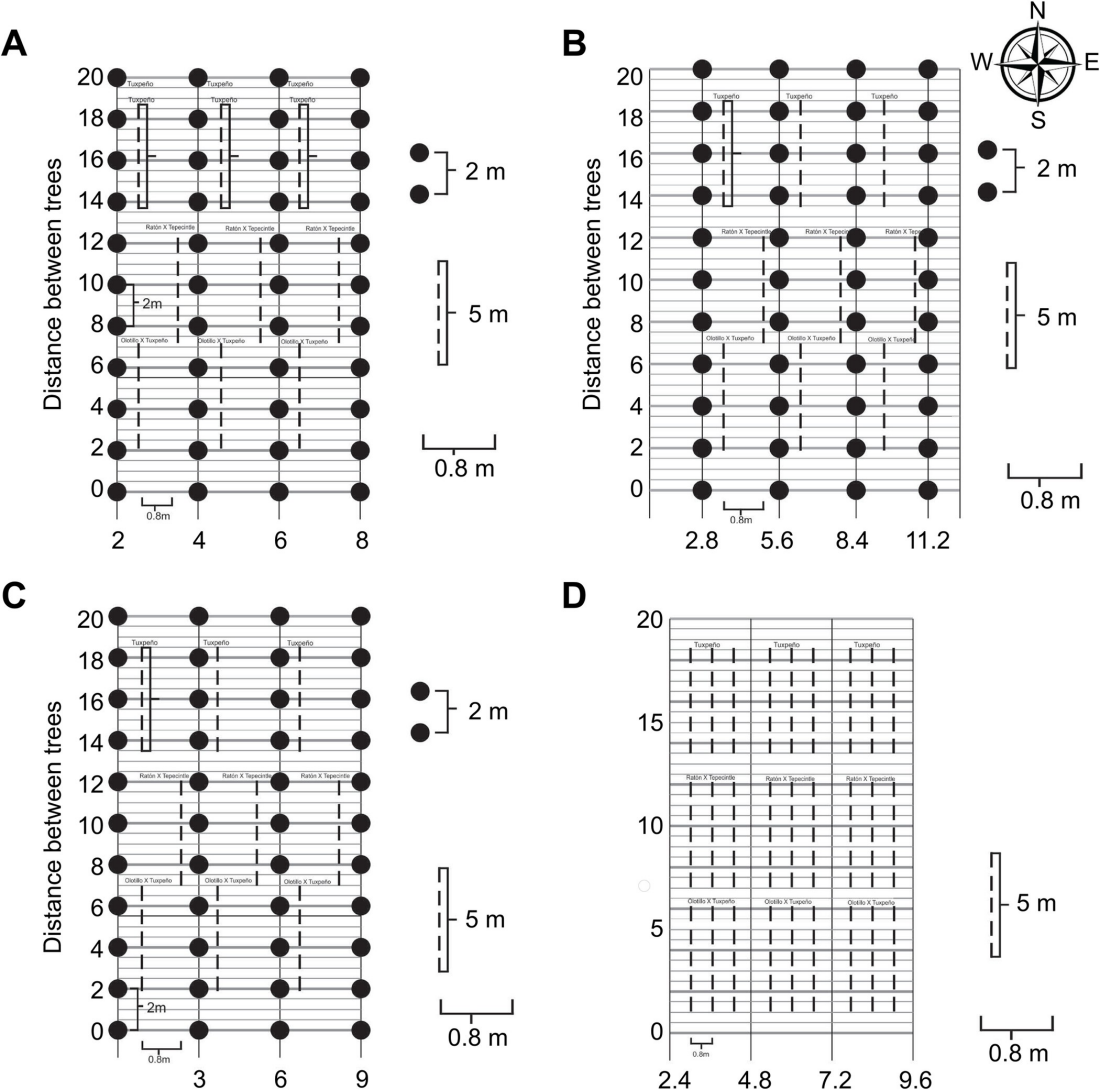
Representation of an Experimental Unit for Each AS: A) *Myroxylon* 2.0 × 2.0, B) *Myroxylon* 2.8 × 2.0, C) *Coffea* 3.0 × 2.0, D) and Native Maize Monoculture. Black circles represent the trees, and discontinuous lines indicate maize plants.

At La Gloria location, three native maize races were planted in a coffee plantation (*Coffea canephora*) aged 2 years, with a plantation arrangement of 3.0 × 2.0 m (1666 plants ha-1). In the La Luisa location, the same races of maize were planted in two balsam ASs (*Myroxylon balsamum* L.*)* in two plantation arrays of 2.8 × 2.0 m (1785 plants ha^-1^) and 2.0 × 2.0 m (2500 plants ha^-1^). The trees were 3 years old. Additionally, three maize races were planted in a monoculture system with a population density of 62 500 plants ha^-1^ (Fig 1). Maize requires cross-pollination, and pollination was not controlled in the present study.

**Fig 2:**
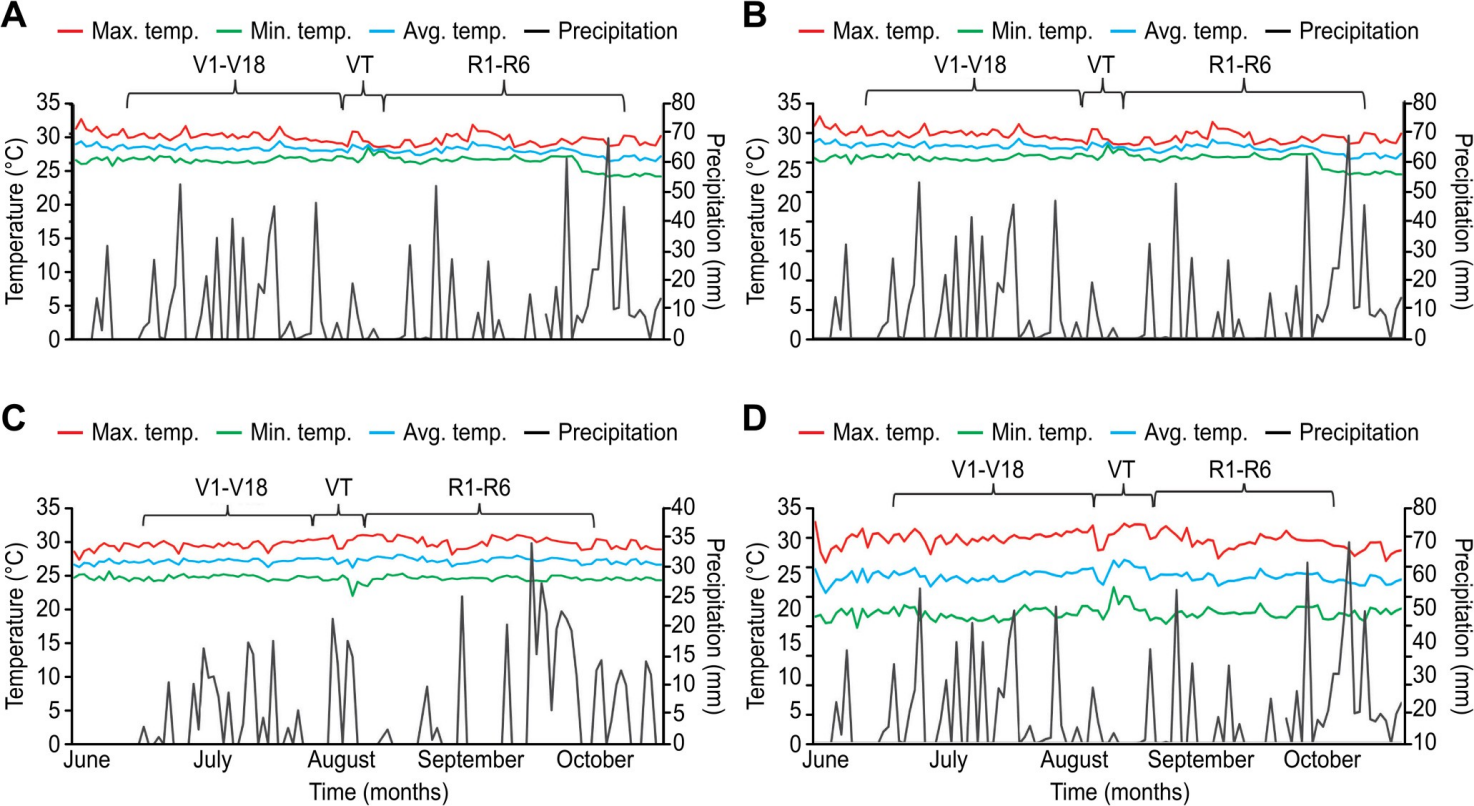
Microclimatic Conditions of Each AS Studied: A) *Myroxylon* 2.0 × 2.0, B) *Myroxylon* 2.8 × 2.0, C) *Coffea* 3.0 × 2.0, D) and Native Maize Monoculture. Phenological stages of the maize plants: V1-V18 (vegetative stage), VT (male flowering), and R1-R6 (reproductive stage), according to Martínez [32].

Culture management was performed according to conventional maize farmer practices in the location. Sowing was performed on 8 and 10 June 2019 under dry farming conditions. During planting with the help of a punch, three seeds were set, i.e., 0.2 m between plants and 0.8 m between ruts. Chemical fertilization was carried out with doses of 46N-00P-00K; one third of the N was applied 25 days after sowing and the remainder during the second weeding. Weeding and cleaning were conducted manually during the crop cycle, and no chemical products were applied to control insects or bushes. For each system, daily temperatures and precipitation were recorded during the whole experiment using a digital thermometer-hygrometer (HTC-2) and a rain gauge with a datalogger (WatchDog 1120) (Fig 2).

### Experimental design and unit

A randomized complete block experimental design with three repetitions was used in a 3 × 4 factorial scheme represented by the three maize races (Factor R) and three agroforestry system arrays (3.0 × 2.0) for *C*. *canephora and M*. *balsamum* at spacings of 2.8 × 2.0 and 2.0 × 2.0 m, respectively, plus monoculture (Factor S), i.e., a total of 12 treatments (Table 2). The experimental unit consisted of 15 maize plants. The morphometric characteristics of the perennial *C*. *canephora and M*. *balsamum* plants heights were 2.1 and 3.0 m, respectively, and the stem diameters were 6.0 cm and 7.0 cm, respectively.

**Table 2:** Description of the evaluated factors of the three maize races in different ASs.

Treatement	Maize race name	System arrangement	Population density (plants ha^-1^)
1	Tuxpeño	Monoculture	62 500
2	Olotillo × Tuxpeño	Monoculture	62 500
3	Ratón × Tepecintle	Monoculture	62 500
4	Tuxpeño	*Coffea* 3.0 × 2.0	33 300
5	Olotillo × Tuxpeño	*Coffea* 3.0 × 2.0	33 300
6	Ratón × Tepecintle	*Coffea* 3.0 × 2.0	33 300
7	Tuxpeño	*Myroxylon* 2.8 × 2.0	35 000
8	Olotillo × Tuxpeño	*Myroxylon* 2.8 × 2.0	35 000
9	Ratón × Tepecintle	*Myroxylon* 2.8 × 2.0	35 000
10	Tuxpeño	*Myroxylon* 2.0 × 2.0	25 000
11	Olotillo × Tuxpeño	*Myroxylon* 2.0 × 2.0	25 000
12	Ratón × Tepecintle	*Myroxylon* 2.0 × 2.0	25 000

### Variables evaluated

In each experimental unit, 10 plants with complete competition were chosen at random in each plot and were labeled to obtain their morpho-agronomic data. Complete competition means that those maize plants that had neighboring ones in the four cardinal points were chosen. The plant height (cm) and stem diameter (mm) were quantified. At harvest, five ears were collected from previously labeled plants, and the length and ear diameter (mm), number of kernels per row, and cob diameter (mm) were recorded. The ears were shelled manually; 10 kernels were collected, the length and width (mm) were determined, and the average weight of 100 kernels was recorded. These seeds were adjusted to 10% humidity. Variables were evaluated according to the maize descriptors of [9, 33, 34].

### Statistical analysis

Data obtained from the morpho-agronomic measures were analyzed by the Shapiro–Wilk test to verify a normal distribution, whereas Levene’s test was used for homogeneity of variance [35]. The morpho-agronomic variables of the maize races studied were analyzed by analysis of variance (ANOVA) under a randomized complete block design. When significant differences were detected between races and between ASs, Tukey’s separation (*p* ≤ 0.05) *post hoc* test was applied. To associate the morpho-agronomic variables of the native maize races with the microclimatic variables of the AS arrangements, principal component analysis (PCA) was conducted with a correlation matrix [36] using the PRINCOMP procedure of SAS v. 9.1 [37] to obtain the eigenvalues and eigenvectors. Based on a graph by Gabriel [38], the correlation structure between variables and the magnitude of each of them relative to the global variation were visualized [39]. Before PCA, the data were standardized to mean 0 (zero) and variance 1. The results are shown in a biplot with the two PCs, as those that presented a minimum cumulative variance of 70% were sought [25] to detect possible association patterns based on similarities-dissimilarities of the morpho-agronomic and microclimatic variables [25, 35]. These analyses were performed with the Statistical Analysis System v. 9.1 [37].

## Results

### Morpho-agronomic variables

The average squares of the morpho-agronomic variables in the native maize races of Mexico are presented in Table 3. According to the ANOVA, significant differences were observed (*p* ≤ 0.05) in the plant height, stem diameter, ear length and width kernel characteristics of the native maize races evaluated for the R and R x S factors. However, for the latter, a significant difference existed at *p* ≤ 0.001 for kernel length and the 100-kernel weight (Table 3). Therefore, there is evidence of a particular adaptation of the races to each of the systems. ANOVA showed a significant difference (*p* ≤ 0.001) in 100% of the analyzed variables for the S factor (Table 3), which suggests an effect on each characteristic evaluated in the present work.

**Table 3:** Means, average squares and coefficient of variation of the ANOVA of the morpho-agronomic variables in native maize races of Mexico under different systems.

Variable (Measurement unit)	Race (R)	System (S)	Race × System (R × S)	Mean	CV (%)
Plant height (cm)	6695.43**	6387.41**	9742.86**	325.7	7.7
Stem diameter (mm)	20.02*	125.00**	28.55**	27.4	7.7
Ear length (cm)	5.02*	66.15**	5.15**	15.1	8.0
Ear diameter (mm)	15.65^ns^	206.73**	11.09 ^ns^	41.4	6.3
Number of kernel rows	2.60 ^ns^	10.06**	1.58 ^ns^	11.9	7.9
Number of kernels per rows	25.40 ^ns^	155.00**	32.11 ^ns^	23.8	17.0
Cob diameter (mm)	15.35 ^ns^	67.17**	13.17 ^ns^	20.8	13.4
Kernel length (mm)	0.62 ^ns^	8.55**	1.08**	10.4	5.1
Kernel width (mm)	4.32**	5.13**	2.03**	9.7	5.2
100-kernel weight (g)	0.02*	4.95**	0.50**	30.0	9.7

* Significance at 0.05

** significance at 0.001; ns: no significant difference CV: coefficient of variation.

**Fig 3:**
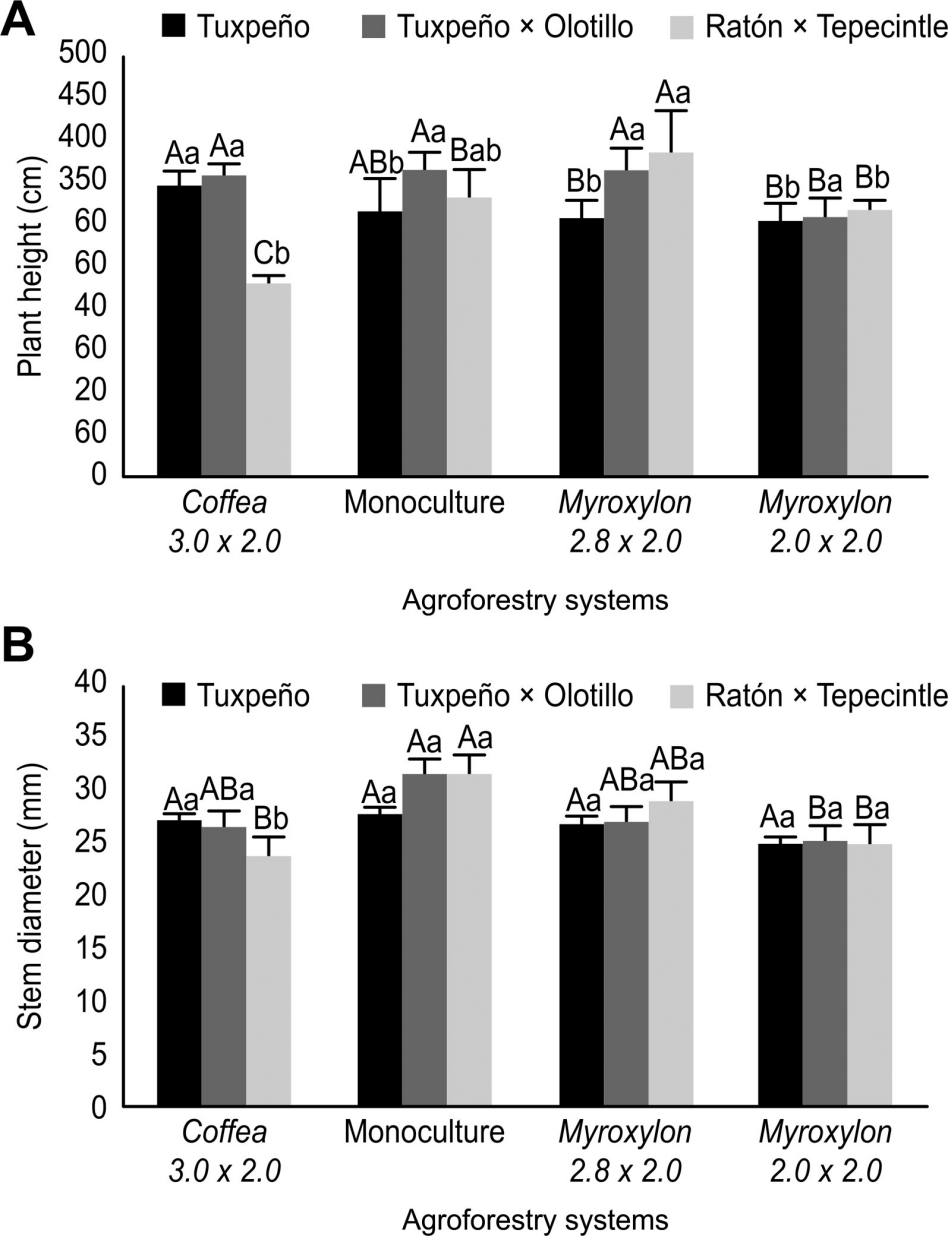
(A) Plant Height and (B) Stem Diameter of Three Native *Z*. *mays* Races (Tuxpeño, Tuxpeño × Olotillo and Ratón × Tepecintle) Associated with AS and Cultivated in Monoculture. The values for each maize race followed by the same lowercase letter do not differ significantly, while the means for each system marked with the same uppercase letter do not differ significantly according to Tukey’s test, *p* ≤ 0.05). The bars represent the standard error of the mean.

The Tuxpeño race associated with *Coffea* bushes had a greater plant height (PH; 343.5 cm; *p* ≤ 0.001) compared with *Myroxylon* 2.8 × 2.0 and 2.0 × 2.0 m, with values of 305.5 and 301.5 cm, respectively (Fig 3A). The PH of the Olotillo × Tuxpeño race in the *Coffea* 3.0 × 2.0 m, *Myroxylon* 2.8 × 2.0 and monoculture systems was statistically similar (*p* ≥ 0.001), with means of 356.3 cm, 362.3 cm and 362.0 cm, respectively, but these systems were different (*p* ≤ 0.001) from AS *Myroxylon* 2.0 × 2.0 m, with 306.5 cm PH (Fig 3A). Moreover, the Ratón × Tepecintle race grown with *Myroxylon* 2.8 × 2.0 was taller (383 cm; *p* ≤ 0.001) than that in the other systems (Fig 3A).

The Tuxpeño race in the three ASs and in monoculture did not present significant differences (*p* ≥ 0.001) for in stem diameter (SD) with means of 27.3 mm (*Coffea* 3.0 × 2.0), 27.9 mm (monoculture), 27 mm (*Myroxylon* 2.8 × 2.0) and 25 mm (*Myroxylon* 2.0 × 2.0) (Fig 3B). Monocultured Olotillo × Tuxpeño had better performance in SD, i.e., 31.2 mm, which was significantly different (*p* ≤ 0.001) from the conditions with *Myroxylon* 2.0 × 2.0 m, with a value of 25.4 mm. The monoculture with the Ratón × Tepecintle race presented the highest SD (31.7 mm; *p* ≤0.001), in contrast to values of 23.8 mm and 25.0 mm, corresponding to the *Coffea* 3.0 × 2.0 m and *Myroxylon* 2.0 × 2.0 environments, respectively (Fig 3B).

**Fig 4:**
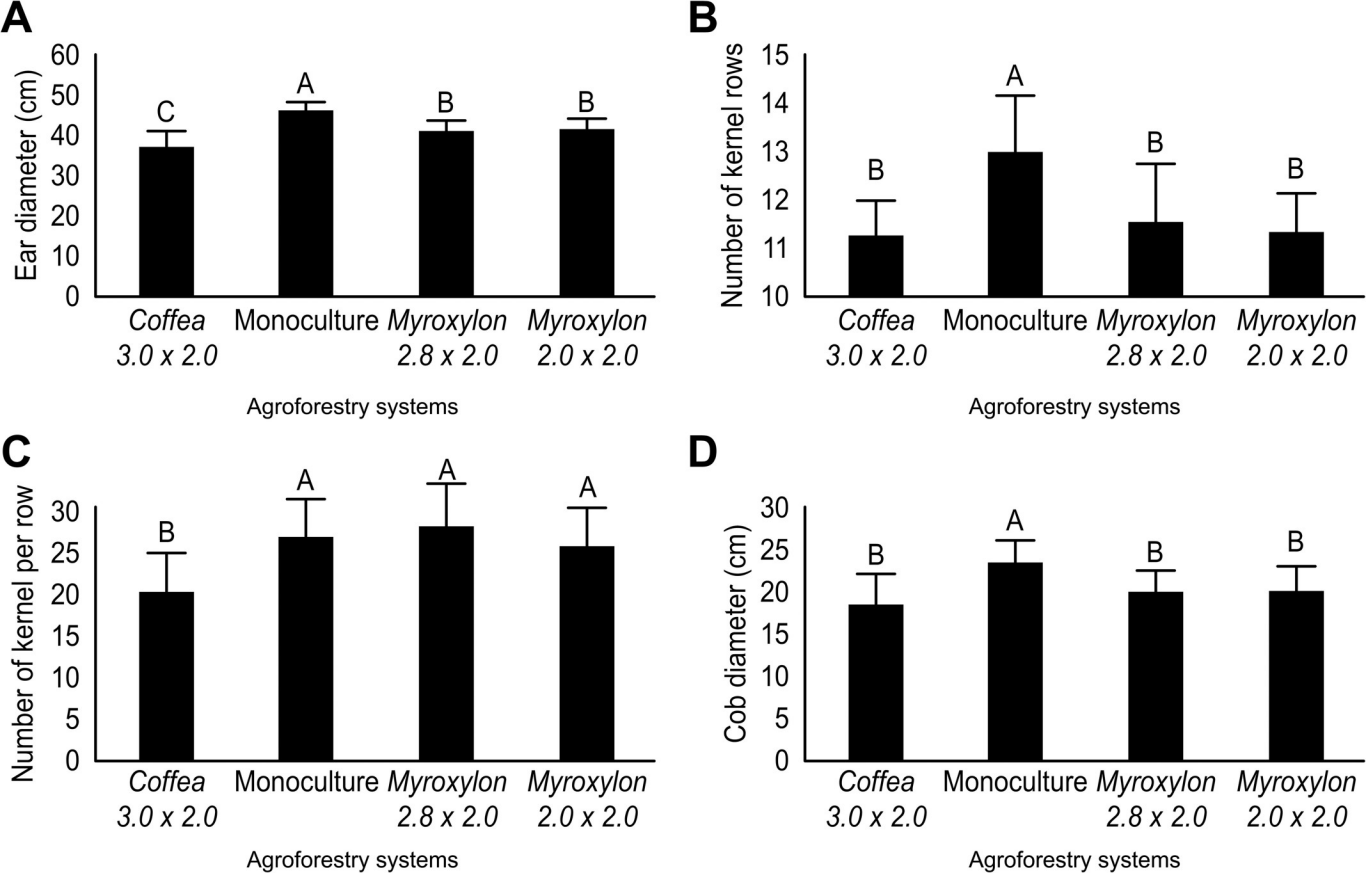
Ear Diameter (A), Number of Kernel Rows (B), Number of Kernels Per Row (C) and Cob Diameter (D) of Three Native Maize Races (Tuxpeño, Olotillo × Tuxpeño and Ratón × Tepecintle) Associated with AS and Cultivated in Monoculture. The values for each maize race followed by the same lowercase letter do not differ significantly, while the means for each system marked with the same uppercase letter do not differ significantly according to Tukey’s test, *p* ≤ 0.05). The bars represent the standard error of the mean.

Plants from the maize races evaluated in this present work had the greatest cob diameter (EarD) (46 mm; *p* ≤ 0.001) in monoculture, compared with the *Coffea* system (3.0 × 2.0 m), which had the lowest value (37 mm) (Fig 4A).

The native maize races evaluated in this study had a greater number of rows on the cob (NKeR) (13.00; *p* ≤ 0.001) under monoculture conditions compared with the AS *Myroxylon* (in the two planting arrays) and *Coffea* 3.0 × 2.0 m, by 11.0, 11.6 and 11.4 rows, respectively (Fig 4B).

The native maize races studied here had 25.0, 26.0 and 24.0 grains per row (NKPR); these values were not significantly different (*p* ≥ 0.001) between the monoculture systems *Myroxylon* 2.8 × 2.0 and *Myroxylon* 2.0 × 2.0 m, but were higher (*p* ≤ 0.001) than the 19.0 value recorded in the culture system with *Coffea* (Fig 4C).

The 23.7 mm cob diameter value (COBD) for the native maize races, evaluated in the monoculture environment, was higher (*p* ≤ 0.001) than those in the arrays of *Coffea* 3.0 × 2.0 m, *Myroxylon* 2.8 × 2.0 m and *Myroxylon* 2.0 × 2.0 m, with means of 18.7, 20.3 and 20.4 mm, respectively (Fig 4D).

**Fig 5:**
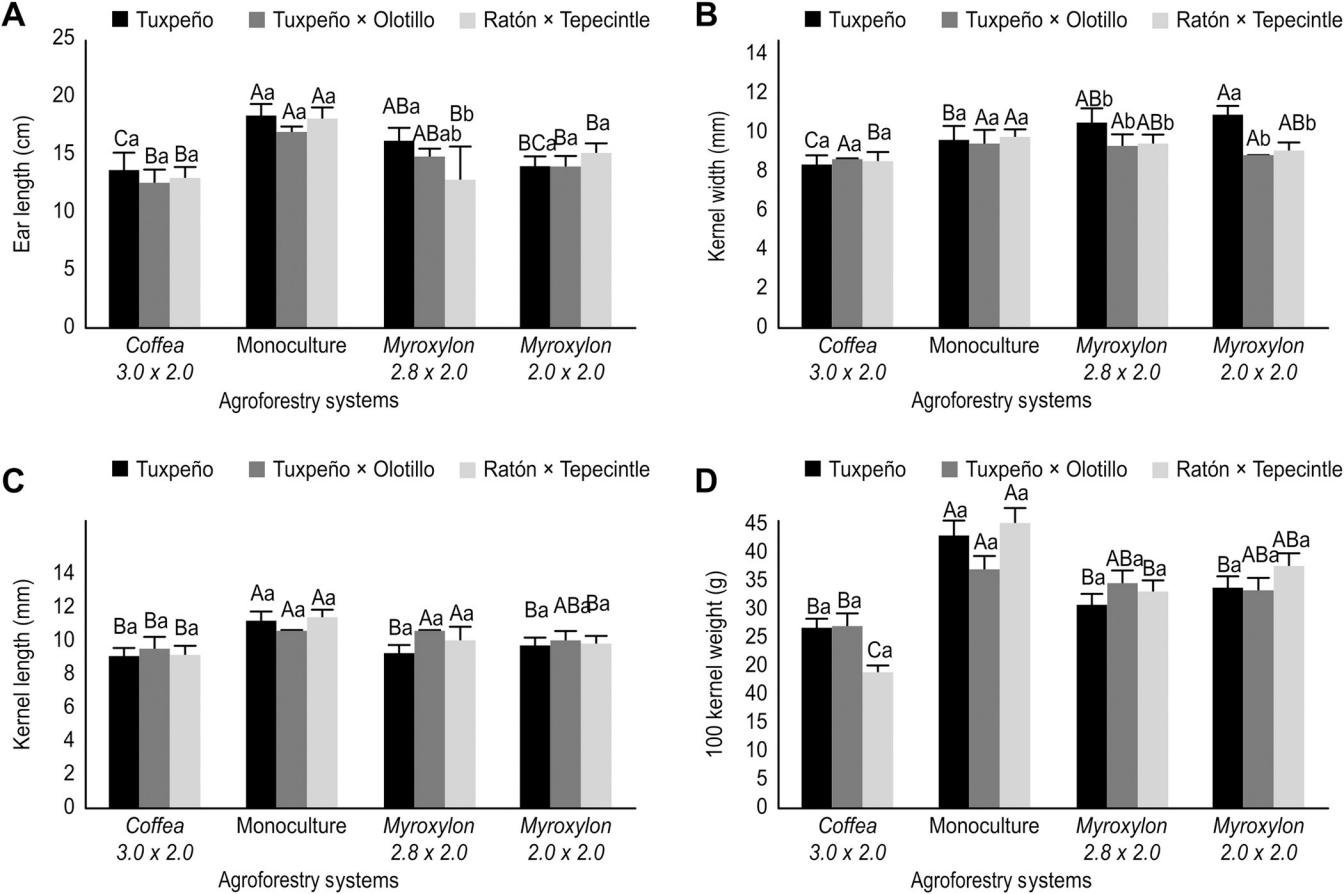
Ear Length (A), Kernel Width (B), Kernel Length (C) and 100-Kernel Weight (D) of Three Native Maize Races (Tuxpeño, Olotillo × Tuxpeño and Ratón × Tepecintle) Associated with AS and Cultivated in Monoculture. The values for each maize race followed by the same lowercase letter do not differ significantly, while the means for each system marked with the same uppercase letter do not differ significantly according to Tukey’s test, *p* ≤ 0.05). The bars represent the standard error of the mean.

The monoculture and *Myroxylon* 2.8 × 2.0 m with the Tuxpeño race did not differ statistically (*p* ≥ 0.05) as they presented means of 18.2 and 16.1 cm for EL, but these were different (*p* ≤ 0.05) from the *Coffea* environment, with a value of 13.5 cm (Fig 5A). The monoculture and *Myroxylon* 2.8 × 2.0 m systems with the Olotillo × Tuxpeño race did not present statistically significant differences (*p* ≥ 0.05), with means of 16.8 and 14.7 cm, respectively. The Ratón × Tepecintle monoculture had the highest EL (18.0 cm; *p* ≤ 0.001) compared with intercropping with AS *Coffea* and *Myroxylon* (Fig 5A).

With respect to the Tuxpeño race in terms of KeWi, no statistically significant differences (*p* ≥ 0.05) were found between the AS *Myroxylon* 2.8 × 2.0 m and *Myroxylon* with 2.0 × 2.0 m arrangement, showing values of 10.7 and 11.1 mm. However, the *Coffea* system had the lowest value (9.8 mm; Fig 5B). The Olotillo × Tuxpeño race did not show significant changes (*p* ≥ 0.001) in the KeWi characteristic among the four systems, *Coffea* 3.0 × 2.0 m, conventional, *Myroxylon* 2.8 m × 2.0 and *Myroxylon* 2.0 × 2.0 m, with values of 8.8, 9.6, 9.5 and 9.0 mm, respectively (Fig 5B). The Ratón × Tepecintle monoculture presented ears with higher KeWi (9.9 mm; *p* ≤ 0.05) than in the *Coffea* arrangement 3.0 × 2.0 m, i.e., 8.8 mm (Fig 5B).

The Tuxpeño collection had greater grain length (KeLe) (11.4 mm; *p* ≤ 0.001) in the monoculture system compared with the means of 9.2, 9.4 and 9.9 mm for AS *Coffea*, *Myroxylon* 2.8 × 2.0 m and *Myroxylon* 2.0 × 2.0 m, respectively (Fig 5C). The Olotillo × Tuxpeño race did not show significant differences (*p* ≥ 0.001) in KeLe when it grew under monoculture, *Myroxylon* 2.8 × 2.0 m and *Myroxylon* 2.0 × 2.0 m, with values of 10.8, 10.8 and 10.2 mm in each culture system (Fig 5C). The monoculture in which the Ratón × Tepecintle race was established presented the best KeLe values (11.6 mm; *p* ≤ 0.001), while smaller means were measured in the maize plants with the *Coffea* 3.0 × 2.0 m, *Myroxylon* 2.8 × 2.0 m and *Myroxylon* 2.0 × 2.0 m arrays (9.3, 10.2 and 10.0 mm, respectively) (Fig 5C).

The Tuxpeño race had a higher value for the weight of 100 kernels (100KW) (38.0 g; *p* ≤ 0.001) under monoculture conditions compared with the ASs *Coffea* (3.0 × 2.0 m), *Myroxylon* (2.8 × 2.0 m) and *Myroxylon* (2.0 × 2.0 m) (25.2, 28.4 and 30.8 g, respectively) (Fig 5D). On the other hand, there are no statistically significant differences in Olotillo × Tuxpeño between the conventional system and *Myroxylon* 2.8 × 2.0 m and *Myroxylon* 2.0 × 2.0 m, which presented means of 33.4, 31.4 and 30.4 g for 100KW (Fig 5D). Between the monoculture systems and *Myroxylon* 2.0 × 2.0 m, no significant differences were detected (*p* ≥ 0.05), in the Ratón × Tepecintle race, with means of 39.8 and 33.9 g for 100KW in each system. However, these values in the monoculture and *Myroxylon* 2.0 × 2.0 m were higher (*p* ≤ 0.05) than the 19.0 g for *Coffea* 3.0 × 2.0 m (Fig 5D).

### Associations between the morpho-agronomic variables of the native maize races and the microclimatic conditions of the agroforestry systems

**Fig 6:**
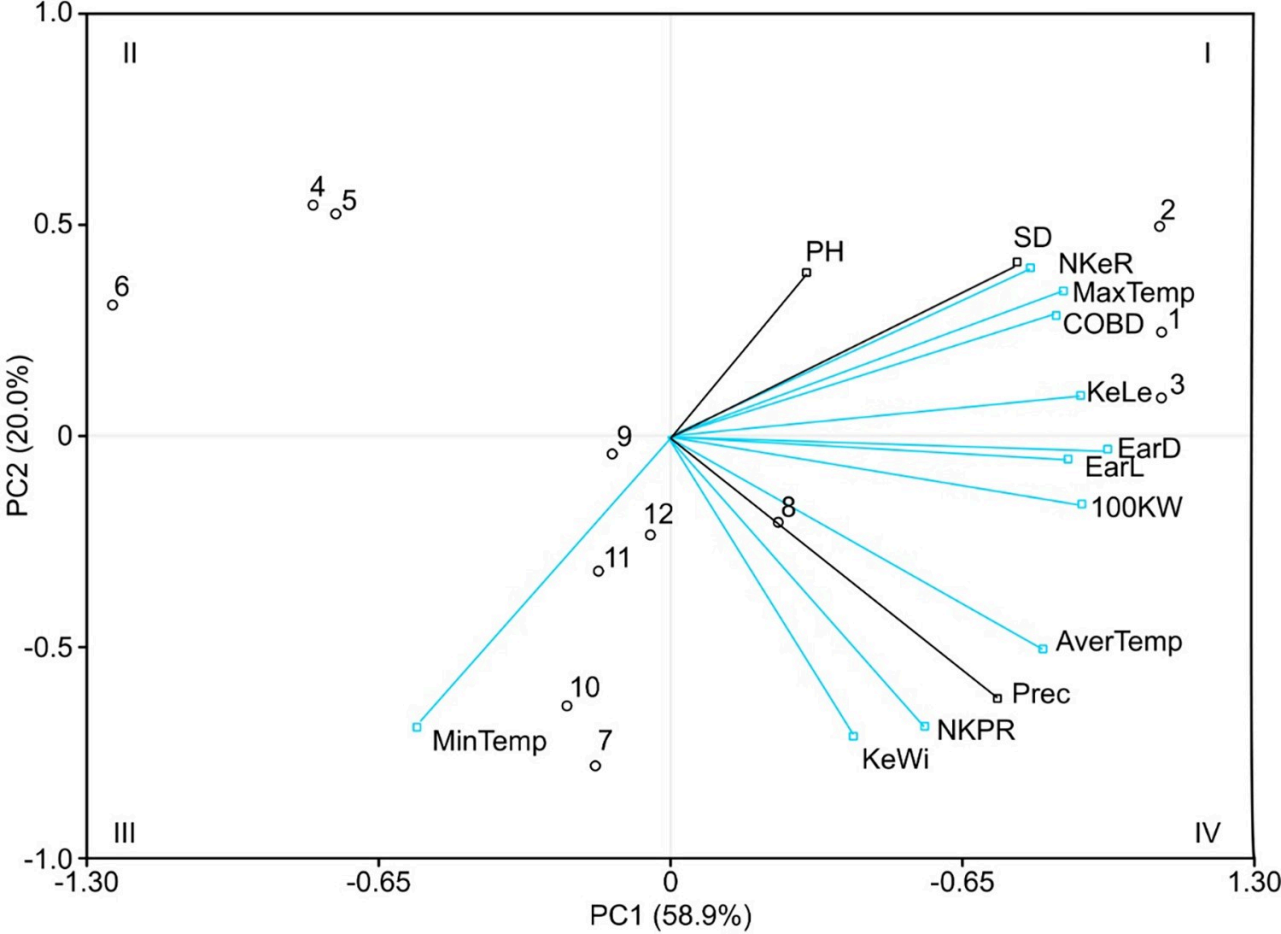
Biplot AS and monoculture (12 treatments) arrangements, built with the first two PCs of 11 agro-morphological and microclimatic variables, which were evaluated in a crop cycle in 2019. For abbreviations and variable numbers, see Table 4. The Arabic numbers represent the treatments; see Table 2. The blue arrows represent the variables that contribute to each PC.

According to the PCA results, there was a positive correlation between the morpho-agronomic variables of the native maize races and the microclimatic conditions that fluctuated depending on the AS and monoculture (Fig 6). The first 11 principal components (PCs) explained 100% of total variance (data not shown). However, the first three PCs explained 87.7% of the total variance between the different SA arrays analyzed (Table 4). According to the method proposed by Pla [39] with a value of 8.2, the variables associated with PC1 were ear diameter, ear length, kernel length, weight of 100 kernels, cob diameter, number of kernel rows, maximum temperature and average temperature, while kernel width, number of kernels per row and minimum temperature were related to PC2 (value of 3.3). With respect to PC3, only plant height contributed, with a value of 1.2 (Table 4).

**Table 4:** Eigenvectors, eigenvalues and proportion of the explained variation for 14 variables evaluated among maize races native to Mexico in ASs based on their principal components (PCs).

Variable (Measurement unit)	Acronym	PC1	PC2	PC3
Plant heigh (cm)	PH	0.11 (0.30)	-0.23 (0.38)	**0.74** (0.82)
Stem diameter (mm)	SD	0.27 (0.77)	-0.25 (0.41)	0.30 (0.32)
Ear length (cm)	EL	**0.31** (0.88)	0.03 (-0.05)	-0.33 (-0.36)
Ear diameter (mm)	EarD	**0.34** (0.97)	0.02 (-0.03)	0.06 (0.06)
Number of kernel rows	NKeR	**0.28** (0.80)	-0.24 (0.39)	-0.10 (-0.12)
Number of kernels per rows	NKPR	0.20 (0.56)	**0.41** (-0.68)	-0.11 (-0.01)
Cob diameter (mm)	COBD	**0.30** (0.85)	-0.17 (0.28)	0.16 (0.17)
Kernel length (mm)	KeLe	**0.32** (0.91)	-0.06 (0.09)	-0.02 (-0.01)
Kernel width (mm)	KeWi	0.14 (0.40)	**0.42** (-0.70)	-0.12 (-0.01)
100-kernel weight (g)	100KW	**0.32** (0.91)	0.09 (-0.16)	0.01 (0.02)
Maximun Temperature	MaxTemp	**0.30** (0.87)	-0.21 (0.34)	-0.26 (-0.28)
Minimun Temperature	MinTemp	-0.20 (-0.56)	**0.41** (-0.68)	0.34 (0.37)
Average Temperature	AverTemp	**0.29** (0.83)	0.30 (-0.50)	0.08 (0.08)
Precipitation	Prec	0.25 (0.72)	0.37 (-0.62)	0.13 (0.15)
Eigenvalue		8.2	2.8	1.2
Variation explained (%)		58.9	20.0	8.7
Cumulative variation (%)		58.9	78.9	87.7

Variable correlation coefficients with the PC are in parentheses. Bold numbers indicate the contribution of the variables to each PC.

The spatial distribution of the association of the different AS arrangements with respect to the morpho-agronomic and microclimatic characteristics was concentrated in quadrants I and IV (Fig 6). PC1 placed treatments 1, 2, 3 and 8 in quadrants I and IV because the three maize races under monoculture conditions and in *Myroxylon* 2.8 × 2.0 m had the highest values for ear length, ear diameter, cob diameter, kernel length, weight of 100 kernels, maximum temperature and average temperature in contrast to the other arrangements of quadrants II and III (Fig 6). In contrast, in PC2, treatments 1, 2, 3, 4, 5 and 6 belonging to the monoculture schemes and *Coffea* 3.0 × 2.0, respectively, were associated with quadrants I and II, and the maize races that had the lowest kernel width values, number of kernels per row and minimum temperature with respect to the *Myroxylon* (2.8 × 2.0 and 2.0 × 2.0 m) treatments were concentrated in quadrants III and IV (Fig 6).

## Discussion

### Morpho-agronomic variables

The objective of this study was to evaluate the variation in the morpho-agronomic characteristics of three maize races, Tuxpeño, Olotillo × Tuxpeño, and Ratón × Tepecintle, native to Mexico cultivated under different AS schemes. The significant differences found among the ASs indicate that the morpho-agronomic characteristics of the native maize races evaluated were affected by the AS arrangement and therefore by the tree canopy, with an impact on biomass production, as shade negatively affects plants with C4 metabolism, such as maize, as it requires more solar radiation for physiological and photosynthetic processes [21]. Although tree shade was not directly evaluated in the present study, it induces a heterogeneous light environment for the crops beneath the canopy [24]. In this sense, it has been documented that 70% shade positively affected the height of two grass species (*Brachiaria decumbens* and *Brachiaria brizantha*), with stem elongation and a greater leaf area with less weight adopted as strategies to intercept as much light as possible, although this did not manifest in an increase in productivity [40].

The native maize races analyzed in this study had particularities in the morpho-agronomic characteristics in each AS even when there were differences between the microclimatic conditions of the AS, particularly temperature (Fig 2). For instance, in general, the plant height and stem diameter of the Tuxpeño race did not present significant differences between the AS *Myroxylon* 2.8 × 2.0 m, *Coffea* 3.0 × 2.0 m and monoculture (Fig 3). In this sense, the planting arrangements of each AS and the plasticity of the characteristics of the maize plants are determined by the variety of environmental conditions that prevail in the AS [22]. For example, Caron et al. [41] found that the high growth rate of *Eucalyptus* reduced the stem weight of *S*. *officinarum* by 50%, attributing this discrepancy to interspecific competition, particularly due to the interception of solar radiation by the crop.

Some variables that are correlated with plant height and stem diameter are the total dry matter and leaf area index [42]. In this regard, Schwerz et al. [21], when intercropping *S*. *officinarum* with *Aleurites fordii* trees, recorded greater dry matter and leaf area index in the 12 × 12 m planting arrangement than in the 6 × 6 m arrangement, so they concluded that the canopy, biology (perennial or deciduous) and age of the tree play an important role in the vegetative stage of the species studied. In addition to planting density, tree species influence the growth of maize hybrids. In this sense, Nardini et al. [23] measured a higher relative growth rate in maize under *Eucalyptus* than under *Peltophorum dubium*.

The ear diameter, number of kernel rows, and cob diameter of the native maize and the Tuxpeño, Olotillo × Tuxpeño, and Ratón × Tepecintle races presented higher values (*p* ≤ 0.05) in monoculture than when intercropped with *Coffea* and *Myroxylon* (Fig 4). The number of kernel rows is a variable considered stable [43]; however, in the present work in agroforestry systems, stability was not maintained because in these environments, the number of kernel rows of the three races of *Z*. *mays* decreased compared to the monoculture system (Fig 4B). This may be due to the microclimatic conditions of each system, since in the monoculture, the average temperature exceeded 28°C (Fig 2). Scherwerz et al. [21] noted that the canopy of the forest species influenced the microclimatic conditions in each system and affected the growth rate of *S*. *officinarum*. Similar results were found by Rocandio-Rodríguez et al. [9], who reported significant differences in the number of kernel rows, ear diameter, and cob diameter in seven native maize races from Mexico. Such differences were attributed to climatic and edaphic contrasts among the three environments. Additionally, these researchers noted that crop management influenced the characteristics of *Z*. *mays*.

The number of kernels per row did not differ significantly (*p* ≥ 0.05) among the three maize races analyzed in the present study in monoculture or *Myroxylon* AS (Fig 4C). Data for this variable are important because grain yield values depend on this variable, as indicated by Pecina-Martínez et al. [43]. Rincón-Tuexi et al. [44] showed that the higher the number of rows, the more kernels there are per ear, which is reflected in the grain yield. In the AS *Myroxylon* 2.8 × 2.0 m and 2.0 × 2.0 m systems, the number of kernels per row was statistically similar to that in the monoculture (Fig 4C), which suggests that adequate yield may be obtained with these plantation densities; however, in future studies it would be desirable to take this variable into account to rule out hypotheses. Purroy-Vásquez et al. [45] reported that the yield of native maize from the State of Veracruz, Mexico, was 0.919 t ha^-1^ under rainfed agricultural conditions. In addition, it can be inferred that the biological characteristics of the *Myroxylon* tree favor this characteristic of the maize races studied in this work.

Generally, the greatest cob diameter and ear length values were measured in the monoculture system, while the lowest values were measured in the AS *Coffea* 3.0 × 2.0 m system, independent of the maize race. These variables may be associated with the different microclimatic conditions of the ASs (Fig 2). By contrast, in a study with seven races of native maize from Mexico, Rocandio-Rodriguez et al. [9] found a significant genotype × environment effect for cob diameter but not ear length.

Pecina-Martínez et al. [43] found significant differences between environments and groups of populations in the total number of grains, number of kernel rows, kernels per row and individual grain weight, suggesting that there is variability and specificity within and between different population groups. It should be noted that the values recorded for the number of rows per ear in our study are within the range of 10 to 14 rows reported by Sierra-Macías et al. [28].

The length and width of the kernel differed significantly among the systems (Fig 5B and 5C), with greater kernel length in the monoculture of the Tuxpeño and Ratón × Tepecintle races, whereas the lowest values were measured in the AS *Coffea* 3.0 × 2.0 m with Olotillo × Tuxpeño. With respect to the kernel width, higher values were found in the *Myroxylon* 2.0 × 2.0 m system with the Tuxpeño race, with a mean of 11.1 mm, while in the monoculture with Ratón × Tepecintle, a high value of 9.9 mm was measured compared with the *Coffea* 3.0 × 2.0 m system. However, Olotillo × Tuxpeño did not show changes in this characteristic among the four systems. These two characteristics of the kernel, among others, have allowed the racial classification of maize from the Highlands of Mexico, as they are the most stable with respect to the environment [9]. However, our findings allow us to report that both the length and width of the kernel are influenced by the prevailing conditions in each AS, although the genotype may play a determining role in the stability of the grain width, as evidenced by the Olotillo × Tuxpeño race.

The weight of 100 kernels of the three native maize races presented higher values (*p* ≤ 0.05) in the monoculture system than in the three established planting schemes. These results show that the grain yield could possibly be affected by the conditions of each system. Although some authors [44] consider that the individual weight of the kernel is a variable little affected by temperature stress, findings from our work reveal that kernel weight was influenced by the planting arrangement differentially between races, possibly due to the microclimatic conditions of each AS. For example, the average temperature in the monoculture was higher than that of the other systems (Fig 2).

Other characteristics, such as yield, of maize hybrids, are affected by the spacing of the plantation and the tree species, so the grain yield was statistically equal for the 1 × 10 m treatment (1000 stalks ha^-1^ of *Gmelina arborea)* in the first two cultivation cycles [20]. The same author emphasized that from the third cycle of cultivation, the control yield exceeded that of treatment 1 since the crown of the tree was closed, leading to both a reduction in available solar radiation and photosynthesis of C4 plants, which are shade intolerant [46].

Finally, in general, it can be summarized that the lowest values of most of the morpho-agronomic variables of the native maize races were measured in the AS *Coffea* system, as they could possibly be affected by secondary metabolites such as alkaloids (caffeine) that are present in the fruits and leaves of coffee bushes, which naturally serve as herbivore repellants [47], and these secondary metabolites may affect the growth of maize plants. In this sense, although the study by Sarvade and Singh [17] did not evaluate the interactions of secondary metabolites between maize and coffee, these researchers documented that the different organs (for example, leaves) of agroforestry plants produce allelochemicals (toxic metabolites) that also affect the growth, germination, development, metabolism, and reproduction of other living organisms, such as weeds, pathogens, insects, and nematodes.

### Associations between the morpho-agronomic traits of the native maize races and the microclimatic conditions of the agroforestry systems

The objective of this study was to associate the morpho-agronomic variables of the three native maize races with the microclimatic conditions recorded in the AS arrays. Gepts [48] points out that the important climatic variables in the development and physiological processes of plants are solar radiation, average air temperature, and average annual rainfall. However, the results obtained in this study show that the morphological and agronomic characteristics of the native maize races were largely related to temperature rather than precipitation, which was demonstrated by PCA (Fig 6), possibly because C4 metabolism requires high amounts of solar radiation for photosynthesis. The results obtained in this study are consistent with those reported by [44] in the sense that temperature significantly influenced the agronomic characteristics of four populations of *Z*. *mays*.

Similar to *Z*. *mays* studied in this work, research related to AS intercropped with annual crops such as *Glycine max* L. Merr. and *Zea mays* L. [46], *Triticum aestivum* L. [19, 22], *Zea mays* L. [20, 23] and *Sacharum officinarum* [21] reported morpho-agronomic and physiological differences in these crops and attributed such important differences to planting density, tree canopy, tree species, tree age, biology (perennial or deciduous), microclimatic conditions, tolerance of shade genotypes, species plasticity and crop management.

Based on the above results, knowledge of the response of the morpho-agronomic variables of native maize races to different planting arrangements with different tree species has increased, and this information will serve public policy decision-makers through the promotion of ASs since they promote sustainable and resilient agriculture in the face of certain biotic or abiotic factors, such as climate change [16].

In addition, rural farmers, by integrating ASs with native annual crops such as maize, will not only continue to conserve a wide diversity of plant genetic resources [27] but will also obtain other products such as wood, grains and ecosystem services, which will contribute to the food security of their families [16, 17]. For this reason, it is advisable to continue with this line of research, since other variables not analyzed in this study must be considered, such as the yield, chemical and physiological characteristics and genetic quality of the native Mexican maize races based on the physical quality (soil chemistry), solar radiation and position of the arrangements (for example, cardinal points) of the agroforestry systems, without excluding aspects of economic profitability [20, 24, 35].

## Conclusions

The morpho-agronomic characteristics of the three described native maize races showed important differences associated with the ASs. The Olotillo × Tuxpeño race stands out, as it did not show significant differences in 50% of the variables analyzed in the monoculture and the *Myroxylon* system with the 2.8 m × 2.0 m arrangement. Therefore, we suggest that wider planting arrangements benefit the morpho-agronomic growth of this maize race. Certain morpho-agronomic characteristics of the maize races studied were influenced by the maximum, average and minimum temperatures, which allowed the differentiation between the different arrangements of AS and monoculture. ASs could be a tool that reduces genetic erosion in the face of the loss of native maize races and in turn contributes to the conservation and sustainable use of germplasm. In addition, they allow diversification, such as grains, wood and ecosystem services.

## Supporting information

S1 Table (DOCX)
