# Supplementary material for: Morpho-agronomic evaluation of native maize races associated with Mexican tropical climate agroforestry systems
Source: PLoS One. 2022 Jun 14;17(6):e0269896. doi: 10.1371/journal.pone.0269896 (PMC9197038; doi:10.1371/journal.pone.0269896)
Supplement: S1 Table — (DOCX) [file pone.0269896.s001.docx]

**Principal components Data**

Eigen Values and proportion of explained and accumulated variance for the fisrt 11 principal components based on variables evaluated among maize races native to Mexico in Agroforestry systems, 2019.

| Principal component | Eigenvalue | Proportion of explained variance | Proportion of accumulated variance |
| --- | --- | --- | --- |
| 1 | 8.2521516 | 58.94 | 58.94 |
| 2 | \| 2.7999238 \| \| --- \| | 20.00 | 78.94 |
| 3 | \| 1.2259128 \| \| --- \| | 8.76 | 87.7 |
| 4 | \| 0.7130303 \| \| --- \| | \| 5.09 \| \| --- \| | 92.79 |
| 5 | \| 0.3451442 \| \| --- \| | 2.47 | 95.26 |
| 6 | \| 0.2978612 \| \| --- \| | 2.13 | 97.39 |
| 7 | \| 0.2290457 \| \| --- \| | 1.64 | \| 99.02 \| \| --- \| |
| 8 | \| 0.0745487 \| \| --- \| | \| 0.53 \| \| --- \| | 99.55 |
| 9 | \| 0.0450465 \| \| --- \| | \| 0.32 \| \| --- \| | 99.88 |
| 10 | \| 0.0163645 \| \| --- \| | 0.12 | 99.99 |
| 11 | \| 0.0009708 \| \| --- \| | 0.01 | 100.00 |
